# Levothyroxine treatment response of cardiometabolic biomarkers in older adults with subclinical hypothyroidism

**DOI:** 10.1210/clinem/dgag155

**Published:** 2026-04-08

**Authors:** Linjun Ao, Raymond Noordam, Stella Trompet, Nicolien A van Vliet, Naveed Sattar, Nicolas Rodondi, Elisavet Moutzouri, Sarah Atighetchi, Patricia M Kearney, Terry Quinn, J Wouter Jukema, Jacobijn Gussekloo, Simon P Mooijaart, Ko Willems van Dijk, Rosalinde K E Poortvliet, Diana van Heemst

**Affiliations:** Department of Human Genetics, Leiden University Medical Center, 2300 RC Leiden, the Netherlands; Department of Clinical Epidemiology, Leiden University Medical Center, 2300 RC Leiden, the Netherlands; Department of Public Health and Primary Care/LUMC Campus The Hague, Leiden University Medical Center, 2333 RC Leiden, the Netherlands; Department of Internal Medicine, Section of Gerontology and Geriatrics, Leiden University Medical Center, 2300 RC Leiden, the Netherlands; Department of Internal Medicine, Section of Gerontology and Geriatrics, Leiden University Medical Center, 2300 RC Leiden, the Netherlands; School of Cardiovascular and Metabolic Health, University of Glasgow, Glasgow G12 8TA, UK; Institute of Primary Health Care (BIHAM), University of Bern, 3012 Bern, Switzerland; Department of General Internal Medicine, Inselspital, Bern University Hospital, University of Bern, 3010 Bern, Switzerland; Institute of Primary Health Care (BIHAM), University of Bern, 3012 Bern, Switzerland; Institute of Primary Health Care (BIHAM), University of Bern, 3012 Bern, Switzerland; Department of General Internal Medicine, Inselspital, Bern University Hospital, University of Bern, 3010 Bern, Switzerland; School of Public Health, University College Cork, Pw31584 Cork, Ireland; School of Cardiovascular and Metabolic Health, University of Glasgow, Glasgow G12 8TA, UK; Department of Cardiology, Leiden University Medical Center, 2300 RC Leiden, the Netherlands; Netherlands Heart Institute, 3501 DG Utrecht, the Netherlands; Department of Internal Medicine, Section of Gerontology and Geriatrics, Leiden University Medical Center, 2300 RC Leiden, the Netherlands; Department of Public Health and Primary Care, Leiden University Medical Center, 2300 RC Leiden, the Netherlands; LUMC Center for Medicine for Older People, Leiden University Medical Center, 2300 RC Leiden, the Netherlands; Department of Internal Medicine, Section of Gerontology and Geriatrics, Leiden University Medical Center, 2300 RC Leiden, the Netherlands; LUMC Center for Medicine for Older People, Leiden University Medical Center, 2300 RC Leiden, the Netherlands; Department of Human Genetics, Leiden University Medical Center, 2300 RC Leiden, the Netherlands; Department of Internal Medicine, Division of Endocrinology, Leiden University Medical Center, 2300 RC Leiden, the Netherlands; Einthoven Laboratory for Experimental Vascular Medicine, Leiden University Medical Center, 2300 RC Leiden, the Netherlands; Department of Public Health and Primary Care, Leiden University Medical Center, 2300 RC Leiden, the Netherlands; LUMC Center for Medicine for Older People, Leiden University Medical Center, 2300 RC Leiden, the Netherlands; Department of Internal Medicine, Section of Gerontology and Geriatrics, Leiden University Medical Center, 2300 RC Leiden, the Netherlands

**Keywords:** levothyroxine, subclinical hypothyroidism, older adults, lipids, cardiometabolic biomarkers

## Abstract

**Context:**

Randomized controlled trials (RCTs) found no cardioprotective effects of levothyroxine therapy in older adults with subclinical hypothyroidism.

**Objective:**

The aim of this study was to assess levothyroxine effects on cardiometabolic biomarkers, which may serve as more sensitive treatment indicators.

**Design:**

Post-hoc analysis using (baseline and 12-month) data from two double-blind RCTs in older adults (≥65 years) with subclinical hypothyroidism.

**Main Outcome Measure(s):**

Cardiometabolic biomarkers included seven clinically relevant lipid measures (apolipoprotein B (ApoB), total cholesterol (Total-C), non-high-density lipoprotein cholesterol (non-HDL-C), remnant cholesterol (RC), low-density lipoprotein cholesterol (LDL-C), HDL-C, and triglycerides (TG)) and 167 standardized metabolomic measures from nuclear magnetic resonance. Analyses were additionally stratified by baseline TSH levels.

**Results:**

Among 286 included participants (48% women; median age 75 [70, 82] years; median baseline TSH 6.44 [5.36, 7.81] mIU/L), 142 were randomized to levothyroxine. Overall, levothyroxine showed no effects on ApoB (−0.03 [95% CI: −0.07, 0.00] g/L), Total-C (−0.17 [−0.34, 0.00] mmol/L), non-HDL-C (−0.15 [−0.31, 0.00] mmol/L), RC (−0.09 [−0.16, −0.01] mmol/L), LDL-C (−0.07 [−0.15, 0.02] mmol/L), and TG (−0.07 [−0.15, 0.01] mmol/L). In participants with baseline TSH ≥10 mIU/L (n = 27), potentially beneficial changes (*P* < .05, but not significant after multiple-testing correction) were observed for all clinically relevant lipids except HDL-C, as well as for ApoB-containing lipoproteins, VLDL size, and fatty acids.

**Conclusion:**

In older adults with subclinical hypothyroidism, levothyroxine treatment showed no effects on cardiometabolic biomarkers, although potentially favorable changes in lipids and lipoproteins were observed for individuals with baseline TSH ≥10 mIU/L.

Subclinical hypothyroidism, which is biochemically characterized by elevated thyroid-stimulating hormone (TSH) concentrations in conjunction with thyroid hormone (T4) levels within the population reference range, is a common condition. The prevalence of subclinical hypothyroidism ranges from 4% to 20% in the adult population, with higher prevalences in women and older individuals (1). It is well known that thyroid hormones play vital roles in development, growth, and metabolism (2). More recently, mounting evidence links thyroid dysfunction to aging processes and the development of age-related diseases, including cardiovascular disease (2, 3).

It remains uncertain whether subclinical hypothyroidism increases the risk of cardiovascular disease in older individuals. Evidence based on individual participant data has shown that subclinical hypothyroidism was associated with an increased risk of coronary heart disease only in those with severe subclinical hypothyroidism, characterized by a TSH concentration of 10 mIU per liter (mIU/L) or higher (4). However, to date, randomized controlled trials (RCTs) have failed to observe any apparent cardioprotective effects of levothyroxine therapy in older adults with subclinical hypothyroidism (5, 6). These null findings regarding the clinical benefits of levothyroxine therapy may reflect methodological challenges inherent in studying clinical endpoints, including the need for prolonged follow-up and large sample sizes to detect treatment effects. Importantly, assessing potential effects of levothyroxine earlier in the disease course, perhaps even before disease development, could provide critical insights. Cardiometabolic biomarkers (eg, lipid profiles or inflammatory markers) may serve as sensitive indicators of treatment response, as changes in these intermediate outcomes could predict long-term clinical cardiovascular risk.

Notably, there is increasing evidence that subclinical hypothyroidism is associated with an unfavorable lipid profile, mainly characterized by elevations of varying magnitude in total cholesterol (Total-C), low-density lipoprotein cholesterol (LDL-C), and triglycerides (TG) (7-10). However, most previous evidence comes from observational studies in the general population, which have shown inconsistent findings. Likewise, clinical trials to date have not consistently shown beneficial effects of levothyroxine treatment on lipid levels in older adults with subclinical hypothyroidism (11).

Beyond conventional lipids, emerging evidence highlights the importance of remnant cholesterol (RC) and non-high-density lipoprotein cholesterol (non-HDL-C) in screening for residual risk of cardiovascular disease (12-14). Apolipoprotein B (ApoB) has been shown to be a more accurate marker of cardiovascular risk than LDL-C (15, 16). Moreover, recent advances in nuclear magnetic resonance (NMR)-based metabolomic platforms have enabled detailed investigation of dynamic lipoprotein metabolism and its potential role in disease pathogenesis (17). Despite these developments, studies evaluating the effects of levothyroxine therapy on a comprehensive lipid profile, including RC, non-HDL-C, and ApoB, and other metabolomic measures, remain scarce.

Therefore, based on two previously-conducted large RCTs (18, 19), we aimed to perform an exploratory post-hoc analysis to investigate the potential effects of levothyroxine treatment on cardiometabolic biomarkers in older adults with subclinical hypothyroidism, and to assess whether any of these effects depend on pretreatment TSH levels. The cardiometabolic biomarkers mainly included seven clinically relevant lipid measures (ApoB, Total-C, non-HDL-C, RC, LDL-C, HDL-C, and TG), and NMR-based metabolomics profiles to explore potential underlying biological mechanisms.

## Materials and methods

### Study design and study population

The present study is based on pooled data from two double-blind RCTs, TRUST (Thyroid hormone Replacement for Untreated older adults with Subclinical hypothyroidism—a randomized placebo-controlled Trial) (20) and IEMO80+ thyroid trial (the Institute for Evidence-Based Medicine in Old Age 80-plus thyroid trial) (21). Detailed descriptions and protocols for the two RCTs have been published previously (20, 21). In brief, both trials recruited community-dwelling participants with subclinical hypothyroidism, defined by elevated TSH levels (4.6 to 19.9 mIU/L) measured on at least two occasions between 3 months and 3 years apart, and free T4 levels within the reference range. TRUST recruited participants aged 65 years and older in the Netherlands, Switzerland, Ireland, and the United Kingdom between April 2013 and May 2015. IEMO80+ recruited participants aged 80 years and older in the Netherlands and Switzerland between May 2014 and May 2017. Both trials shared a near-identical design and recruitment strategy. Trial protocols were approved by the relevant ethics committees and regulatory authorities in all countries involved in the trials. Both trials were conducted in accordance with the principles of the Declaration of Helsinki and Good Clinical Practice guidelines. Written informed consent was obtained from all participants (clinical trial registration: ClinicalTrials.gov NCT01660126 [TRUST], Netherlands Trial Register NTR3851 [IEMO80+]).

If participants met the inclusion criteria and agreed to join the trials, they completed the *trial baseline* information. Then, the first visit would take place either at the GP or at the local hospital Clinical Research Facilities, or at the home of the included participants. During the first visit (20, 21), blood samples (40 mL venous blood) for the corresponding biobanks (TRUST biobank and IEMO80+ biobank) were collected, which was defined as the *biobank baseline* in the present study. The timelines of the RCTs and the collection of blood samples for the combined TRUST and IEMO biobanks are presented in Fig. S1 (22). In a previous study using data from these trials, fluctuations in TSH levels collected in routine clinical care were observed to occur over time, showing that 39.9% of participants in the placebo group had normalized TSH concentrations (<4.6mIU/L) within a year (23). Considering the high likelihood of potential fluctuations during the interval between trial baseline and biobank baseline (24), we restricted our study to participants with biochemical subclinical hypothyroidism at the biobank baseline.

Both biobanks comprised plasma and serum samples from all randomized participants, who provided consent for storing biobank materials at the Department of Clinical Chemistry of Leiden University Medical Center (LUMC), the Netherlands. Recruited participants were randomly assigned in a 1:1 ratio to a levothyroxine or placebo group, with titration of the levothyroxine dose according to the TSH level every 6 to 8 weeks and a mock titration schedule with a similar frequency in the placebo group. Participants were followed up for 12 months, and a small blood sample (10 mL venous blood) was taken again at this time. Subsequently, metabolic biomarkers and TSH were measured based on blood samples collected at the Biobank baseline and at the 12-month follow-up.

### Metabolic biomarkers

All metabolic biomarkers investigated in the present study were measured by high-throughput NMR spectroscopy, generated by Nightingale Health Plc (Helsinki, Finland). The Nightingale Health NMR platform has been broadly applied in large-scale epidemiological studies, providing additional evidence to reveal underlying biological mechanisms (17, 25), and showed potential to improve existing prediction models (26).

From these biomarkers, seven established lipid-related biomarkers collected at biobank baseline and 12 months of follow-up were first investigated in this study, including ApoB (g/L), Total-C (mmol/L), non-HDL-C (mmol/L), RC (mmol/L), LDL-C (mmol/L), HDL-C (mmol/L), and TG (mmol/L).

Subsequently, this study further investigated the metabolomic profiles, which included a total of 167 metabolomic measures, comprising amino acids, ketone bodies, glycolysis metabolites, and lipoprotein subclasses varied in size, density, and composition. For each lipoprotein subclass, the lipid concentrations and composition were measured in terms of triglycerides, phospholipids, total cholesterol, cholesterol esters, and free cholesterol, and total lipid concentration within the subclass.

### Statistical analysis

Baseline characteristics of the study population are presented as mean (standard deviation, SD) or median (interquartile range, IQR) for continuous variables and as frequency (proportion) for categorical variables. The effect of levothyroxine therapy on each metabolic biomarker at the 12-month posttreatment follow-up was assessed by an analysis of covariance (ANCOVA) model, with adjustments for the corresponding pretreatment (baseline) metabolic biomarkers. The estimates of treatment effects were presented as *β* coefficients and 95% confidence intervals (95% CI).

The main statistical analyses were restricted to all participants with biobank baseline TSH ≥4.6 mIU/L. Based on previously suggested TSH thresholds for considering levothyroxine treatment in older individuals (27), the ANCOVA model was then repeated in three subgroups: participants with 4.6 ≤ TSH <7 mIU/L, those with TSH ≥7 and TSH <10 mIU/L, and those with TSH ≥10 mIU/L, respectively. The ANCOVA model was applied not only to the seven lipid-related biomarkers, of which TG was log-transformed, but also to the 167 metabolomic measures that were natural log-transformed and standardized before analyses.

Sensitivity analyses were conducted to assess the robustness of the results. First, consistent with previous studies (5, 18), the above primary ANCOVA model was additionally adjusted for sex, age, and start dose of the intervention. Second, sensitivity analyses were performed in the expanded trial baseline sample, which also includes participants who were excluded at the biobank baseline due to having a normalized TSH concentration but previously had a TSH ≥4.6 mIU/L at trial baseline, thereby maintaining alignment with the original RCT designs. Then, to account for the potential effects of taking lipid-lowering medications at baseline (ie, statins, identified by ATC code [C10AA] or corresponding drug names), the interaction between lipid-lowering medication use and levothyroxine treatment was evaluated. Subsequently, stratified analyses were performed, with the primary ANCOVA model being repeated separately for individuals who were and were not taking lipid-lowering medications at baseline. Additionally, to strengthen our findings and leverage the standardized metabolites, which allow for comparison on the same scale, we conducted independent samples t-tests to compare the relative changes of the 167 standardized metabolomic measures from baseline to follow-up between the placebo and levothyroxine groups.

Considering the high intercorrelations between the metabolic biomarkers, the method by Li et al (28) was used to estimate the effective number of independent tests for multiple-testing correction. Therefore, a *P* value below .0125 (0.05/4, with 4 being the effective number of independent tests) was considered statistically significant when analyzing the seven lipid-related biomarkers, and a *P* value below 1.79e−3 (0.05/28, with 28 being the effective number of independent tests) when analyzing the 167 standardized metabolomic measures. All statistical analyses were performed using R software (version 4.3.1).

## Results

The detailed inclusion criteria are presented in Fig. S2 (22). From the pooled TRUST and IEMO80+ Biobanks, 449 participants with both baseline and follow-up data were initially considered. After excluding those with missing lipid-related biomarker or TSH data, as well as those with normalized TSH levels, a total of 286 participants with biochemical subclinical hypothyroidism (48% women, a median age of 75 [IQR: 70, 82] years) were included in the main analysis. Of these, 142 (49.7%) participants were randomized to levothyroxine, and 144 participants to placebo.

As shown in Table 1, the baseline concentrations of the seven lipid-related biomarkers were similar in the levothyroxine and placebo groups. The median [IQR] baseline levels of TSH in the pooled study sample were 6.44 [5.36, 7.81] mIU/L. Among the included participants, 178 (62.2%) had baseline TSH levels above or equal to 4.6 and below 7 mIU/L, 81 (28.3%) had baseline TSH levels above or equal to 7 and below 10 mIU/L, 27 (9.4%) had baseline TSH levels above or equal to 10 mIU/L. In addition, the baseline characterizes stratified by TSH levels are presented in Table S1 (22). Specifically, the group with TSH ≥ 10 mIU/L had the lowest mean (SD) free T4 level at 11.91 (2.49) picomoles per liter (pmol/L), compared to 13.93 (1.89) pmol/L and 13.67 (2.02) pmol/L in the other two TSH groups. The concentrations of the seven lipid-related biomarkers were higher in the group with TSH ≥10 mIU/L compared to the other two TSH groups (Table S1 (22)).

**Table 1:** Baseline characteristics of the included participants in placebo and treatment groups

	Overall	Placebo	Levothyroxine
**N**	286	144	142
**Age (years), median [IQR]**	75 [70, 82]	77 [71, 83]	74 [70, 80]
**Sex = Women, n (%)**	137 (48%)	72 (50%)	65 (46%)
**Site, n (%)**			
Ireland	27 (9.4%)	12 (8.3%)	15 (10.6%)
Netherlands	148 (51.7%)	76 (52.8%)	72 (50.7%)
Scotland	56 (19.6%)	32 (22.2%)	24 (16.9%)
Switzerland	55 (19.2%)	24 (16.7%)	31 (21.8%)
**Ethnics, n (%)**			
Other	4 (1.4%)	3 (2.1%)	1 (0.7%)
White	282 (98.6%)	141 (97.9%)	141 (99.3%)
**Living status, n (%)**			
Co-habiting	174 (60.8%)	90 (62.5%)	84 (59.2%)
Living alone	108 (37.8%)	53 (36.8%)	55 (38.7%)
Other	4 (1.4%)	1 (0.7%)	3 (2.1%)
**Study, n (%)**			
IEMO80+	39 (14%)	24 (17%)	15 (11%)
TRUST	247 (86%)	120 (83%)	127 (89%)
**Start dose, n (%)**			
25 μg	47 (16%)	23 (16%)	24 (17%)
50 μg	239 (84%)	121 (84%)	118 (83%)
**Statin use = Yes, n (%)**	113 (44%)	53 (41%)	60 (47%)
**ApoB (g/L), mean (SD)**	0.89 (0.25)	0.90 (0.26)	0.88 (0.24)
**Total-C (mmol/L), mean (SD)**	4.80 (1.19)	4.86 (1.20)	4.75 (1.17)
**Non-HDL-C (mmol/L), mean (SD)**	3.50 (1.06)	3.54 (1.09)	3.45 (1.03)
**RC (mmol/L), mean (SD)**	1.58 (0.51)	1.60 (0.52)	1.57 (0.51)
**LDL-C (mmol/L), mean (SD)**	1.91 (0.56)	1.94 (0.59)	1.88 (0.54)
**HDL-C (mmol/L), mean (SD)**	1.31 (0.38)	1.33 (0.36)	1.29 (0.39)
**Total-TG (mmol/L), median [IQR]**	1.42 [1.07, 1.88]	1.38 [0.99, 1.98]	1.45 [1.10, 1.85]
**Baseline TSH levels, n (%)**			
4.6 ∼ 7 mIU/L	178 (62.2%)	87 (60.4%)	91 (64.1%)
7 ∼ 10 mIU/L	81 (28.3%)	45 (31.3%)	36 (25.3%)
≥ 10 mIU/L	27 (9.4%)	12 (8.3%)	15 (10.6%)
**Baseline TSH, mIU/L**			
mean (SD)	7.10 (2.71)	7.05 (2.75)	7.15 (2.67)
median [IQR]	6.44 [5.36, 7.81]	6.27 [5.34, 7.81]	6.51 [5.41, 7.72]
Range	4.60 ∼ 24.24	4.60 ∼ 24.24	4.65 ∼ 21.19
**fT4 (pmol/L), mean (SD)**	13.67 (2.07)	13.78 (2.07)	13.56 (2.06)
**fT3 (pmol/L), mean (SD)**	4.37 (0.63)	4.34 (0.59)	4.39 (0.67)

Abbreviations: ApoB, apolipoprotein B; IQR, interquartile range; LDL-C, low-density lipoprotein cholesterol; non-HDL-C, non-high-density lipoprotein cholesterol; pmol/L, picomoles per liter; RC, remnant cholesterol; SD, standard deviation; TG, triglycerides; Total-C, total cholesterol; TSH, thyroid-stimulating hormone.

In addition, 228 participants with complete data for all 167 metabolomic measures at both baseline and follow-up, without any missing values, were included in the metabolomic analysis (Fig. S2 (22)). Of these, 111 (48.7%) were randomized to levothyroxine. The baseline characteristics of the 228 participants (Table S2 (22)) were similar to those included in the analyses of the seven lipids, and the baseline characteristics of the metabolomic profiles are presented in Table S3 (22).

### Effects of levothyroxine treatment on ApoB (g/L) and lipids (mmol/L)

**Figure 1:**
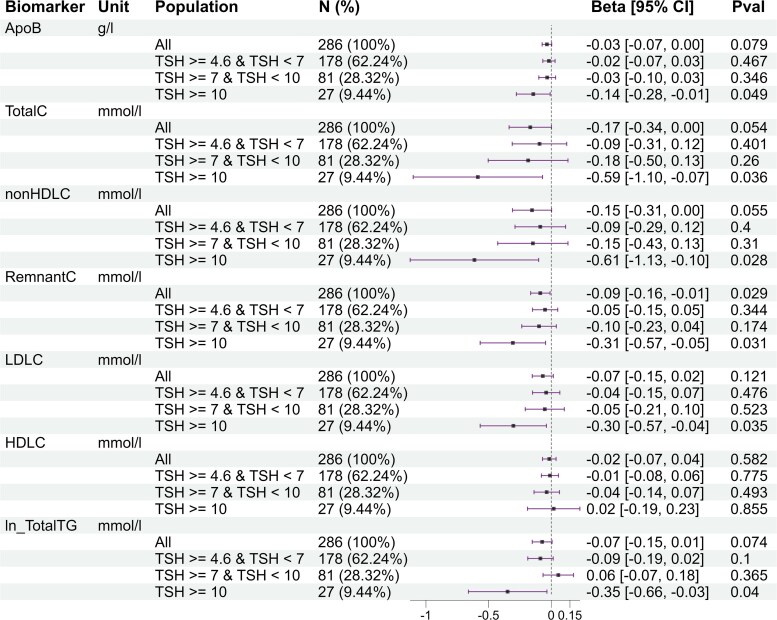
The effects of levothyroxine treatment on lipid-related biomarkers for all included participants and TSH-stratified groups. This figure shows the estimated effects (β and 95% confidence interval) based on the primary ANCOVA models for the corresponding metabolic biomarker in different population groups. The “Pval” column indicates the *P-*values of tests for the estimated associations in the corresponding population group.

The effects of levothyroxine treatment (compared to placebo) assessed by the main ANCOVA model are presented in Fig. 1. For all included participants, after correction for multiple testing, levothyroxine treatment showed no significant effect on the investigated lipid concentrations. Specifically, the mean effects were −0.03 [95% CI: −0.07, 0.00] g/L for ApoB, −0.17 [−0.34, 0.00] mmol/L for Total-C, −0.15 [−0.31, 0.00] mmol/L for non-HDL-C, −0.09 [−0.16, −0.01] mmol/L for RC, −0.07 [−0.15, 0.02] mmol/L for LDL-C, and −0.07 [−0.15, 0.01] mmol/L for natural log-transformed TG. In addition, in the TSH-stratified analyses (Fig. 1), no significant treatment effects were observed after multiple-testing correction, but nominally-significant (*P* < .05) potentially beneficial effects were observed in the subgroup with baseline TSH ≥10 mIU/L across all lipids except HDL-C.

**Figure 2:**
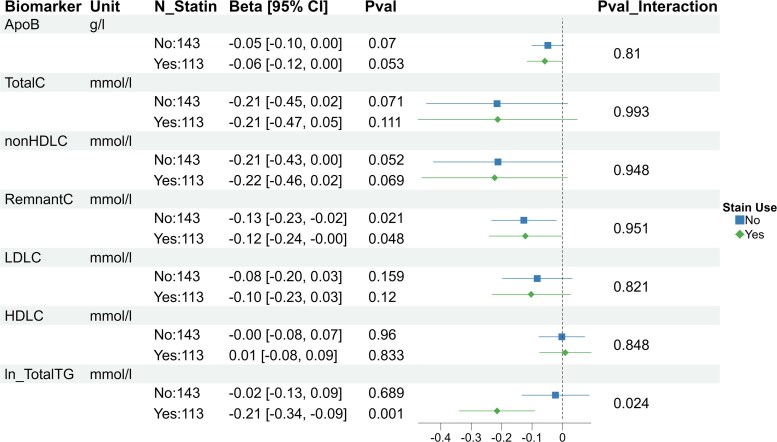
The effects of levothyroxine therapy on metabolic biomarkers in a subgroup stratified by baseline statin use. The “N_Statin” column shows the number of participants without (No) and with (Yes) taking statins at baseline. The “Beta [95% CI]” column indicates the estimated β and 95% confidence interval in subgroups not taking statins and those taking statins at baseline, respectively. The “Pval” column indicates the *P*-values of tests for the estimated associations in subgroups not taking statins and those taking statins at baseline, respectively. The “Pval_Interaction” column shows the results of the interaction tests between levothyroxine treatment and baseline statin use.

Sensitivity analyses, including additional adjustment for other covariates (Fig. S3 (22)) and analyses in the expanded trial baseline sample (Table S4 (22)), both showed results similar to those observed in the main analyses. Furthermore, as shown in Fig. 2, no significant interactions between levothyroxine treatment and baseline statin use were observed among all participants, except for an interaction (*P-value* = .02) for natural log-transformed TG, with an effect of −0.02 [−0.13, 0.09] for participants not taking statins and −0.21 [−0.34, −0.09] for those taking statins.

### Effects of levothyroxine therapy on standardized metabolomic measures

**Figure 3:**
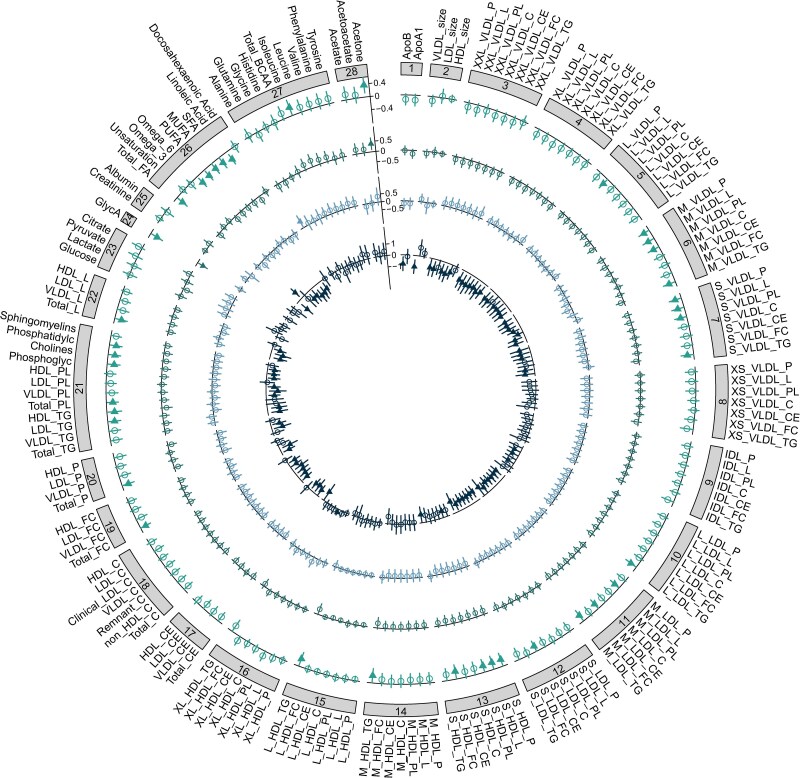
The effects of levothyroxine therapy on standardized metabolomic measurements for all included participants and TSH-stratified groups. From outer to inner circles, each circle represents the estimated *β* and 95% confidence interval based on the ANCOVA models for all participants (n = 228), participants with baseline TSH ≥ 4.6 and TSH < 7 mIU/L (n = 143), participants with baseline TSH ≥ 7 and TSH < 10 mIU/L (n = 62), participants with baseline TSH ≥ 10 mIU/L (n = 23), respectively. Hollow dots, solid triangles, and solid diamonds indicate estimates with *P* > .05, estimates with *P* < .05, and estimates with *P* < 1.79e-3, respectively. Numbers indicate groups: (1) Apolipoproteins; (2) Lipoprotein particle sizes; (3) Extremely large VLDL; (4) Very large VLDL; (5) Large VLDL; (6) Medium VLDL; (7) Small VLDL; (8) Very small VLDL; (9) IDL; (10) Large LDL; (11) Medium LDL; (12) Small LDL; (13) Small HDL; (14) Medium HDL; (15) Large HDL; (16) Very large HDL; (17) Cholesteryl esters; (18) Cholesterol; (19) Free cholesterol; (20) Lipoprotein particle concentrations; (21) Glycerides and phospholipids. (22) Total lipids; (23) Glycolysis-related metabolites; (24) Inflammation; (25) Fluid balance; (26) Fatty acids; (27) Amino acids; (28) Ketone bodies.

Based on the ANCOVA models, the effects of levothyroxine therapy on standardized metabolomic measurements are presented in Fig. 3. For all participants, levothyroxine therapy showed effect directions toward increasing acetone (*P-value* = .003), and decreasing most of the fatty acids (*P* < .05), and a few glycerides and phospholipids (*P* < .05), but these effects were not significant after multiple-testing correction. Similar to the above findings for common lipids, in the subgroup with baseline TSH ≥10 mIU/L, levothyroxine treatment showed nominally-significant (*P* < .05) potentially beneficial effects on lowering levels of ApoB-containing lipoproteins, including LDL and VLDL of varying sizes and compositions, VLDL-size (effect size: −0.98 [95% CI: −1.57, −0.39]), and fatty acids. No evidence was observed in the other two TSH-stratified subgroups.

Sensitivity analyses, including additional adjustment for other covariates (Fig. S4 (22)) and analyses in the expanded trial baseline sample (Fig. S5 (22)), both showed results similar to those of the main analyses. No significant interaction effects between levothyroxine treatment and statin use were observed for all included participants.

**Figure 4:**
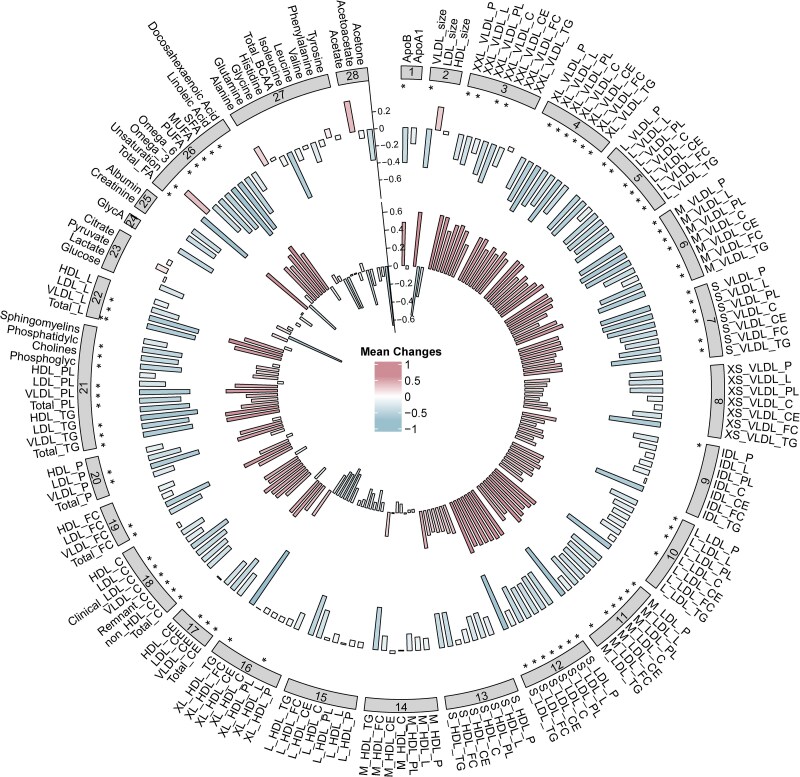
The mean changes from baseline to 12-month follow-up in the standardized metabolite levels for participants with baseline TSH ≥ 10 mIU/L (n = 23). From the outer to inner circles, each bar represents the mean changes of the corresponding metabolite in the levothyroxine and placebo groups, respectively. “**” and “*” indicate tests with *P* < 1.79e-3, and tests with *P* < .05, respectively. Numbers indicate groups: (1) Apolipoproteins; (2) Lipoprotein particle sizes; (3) Extremely large VLDL; (4) Very large VLDL; (5) Large VLDL; (6) Medium VLDL; (7) Small VLDL; (8) Very small VLDL; (9) IDL; (10) Large LDL; (11) Medium LDL; (12) Small LDL; (13) Small HDL; (14) Medium HDL; (15) Large HDL; (16) Very large HDL; (17) Cholesteryl esters; (18) Cholesterol; (19) Free cholesterol; (20) Lipoprotein particle concentrations; (21) Glycerides and phospholipids. (22) Total lipids; (23) Glycolysis-related metabolites; (24) Inflammation; (25) Fluid balance; (26) Fatty acids; (27) Amino acids; (28) Ketone bodies.

The comparison of relative changes from baseline to follow-up in the 167 standardized metabolomic measurements between the placebo and levothyroxine groups (Fig. 4; Figs. S6-S8 (22)) was consistent with the main results from the ANCOVA models. Only in the subgroup with baseline TSH ≥10 mIU/L (Fig. 4), potential main differences (*P* < .05) were observed in the VLDL size, ApoB-containing lipoproteins, and fatty acids, which decreased in the levothyroxine group but increased in the placebo group.

## Discussion

Using exploratory post-hoc analyses of data from two previously-conducted double-blind RCTs (TRUST and IEMO 80+ thyroid trial), we assessed the response of cardiometabolic biomarkers to levothyroxine treatment in older adults with subclinical hypothyroidism. Levothyroxine treatment showed no lipid-lowering benefit in the general older population with subclinical hypothyroidism. However, in the subgroup with baseline TSH ≥10 mIU/L, levothyroxine treatment was nominally associated (*P* < .05, but not significant after Bonferroni correction) with potentially favorable and directionally consistent changes in lipids, including lower concentrations of ApoB, Total-C, non-HDL-C, RC, LDL-C, and TG. Similarly, metabolomic analyses also indicated potentially favorable directionally-consistent alterations in lipoprotein metabolism in this subgroup, with nominal reductions observed in ApoB-containing lipoproteins, VLDL size, and fatty acids.

Previous studies indicated that levothyroxine treatment could improve the lipid profile for individuals with overt hypothyroidism, with pronounced effects on lowering Total-C, LDL-C, and ApoB (29). However, the efficacy of levothyroxine treatment on the lipid profile in people with subclinical hypothyroidism is less clear, especially in the context of different population characteristics and sample sizes. Some meta-analyses of RCTs showed levothyroxine effects on the reduction of Total-C and LDL-C (29-31), and one study also showed lipid-lowering effects in a subgroup with mild subclinical hypothyroidism (TSH <10 mIU/L) (31). Although our study did not observe any significant effects for Total-C or other lipids in the full study population, the observed direction of estimates was consistent with those observed in previous studies. The observed null effects in the present study could be due to (a combination of) modest effect sizes and a relatively small sample size. In addition, prior studies largely focused on conventional lipids (LDL-C, Total-C), but our study incorporating other lipid biomarkers, expanded the evidence by showing a nominally significant effect on lowering RC, a biomarker of residual cardiovascular risk. Our findings for RC, as well as suggestive evidence for other lipids, are supported by a previous report showing significantly decreased levels of TC, non-HDL-C, RC, and ApoB after levothyroxine treatment for people with subclinical hypothyroidism (32).

To the best of our knowledge, our study is the first exploratory study based on post-hoc analysis of data from previous RCTs to investigate the effects of levothyroxine treatment on metabolomic profile in older adults. Despite a lack of statistical significance, the present study observed directional consistency in the potential effects of levothyroxine treatment, with trends toward decreased levels of fatty acids, VLDL size, and ApoB-containing lipoproteins. These findings are consistent with a previous Mendelian randomization study, which showed that genetically-determined higher TSH levels were associated with an unfavorable lipid profile, especially with larger VLDL size and higher levels of TG-rich lipoproteins (7). An important function of thyroid hormone is to stimulate the mobilization, breakdown, and clearance of cholesterol as well as de-novo fatty acids synthesis in the liver (33). Even modest changes in thyroid status may result in a different balance between cholesterol synthesis and clearance (33). Except for effects on cholesterols, a previous study suggested that the observation of higher levels of TG-rich lipoprotein in participants with subclinical hypothyroidism might be related to an increased production of TG-rich lipoproteins, especially large VLDL particles, in the liver (34, 35).

Furthermore, based on the lipid and metabolomic analyses from our study and previous evidence, these findings suggest that traditionally studied lipids, such as LDL-C, may not accurately predict cardiovascular risk in older adults with subclinical hypothyroidism. Instead, RC, a direct marker of TG-rich lipoproteins, and non-HDL-C, which includes TG-rich and other atherogenic lipoproteins, appear to be more relevant indicators for assessing cardiovascular risk associated with subclinical hypothyroidism.

A prior pooled analysis, based on the same TRUST and IEMO80+ thyroid trial, observed no clear clinical advantage of levothyroxine on cardiovascular outcomes in older adults with subclinical hypothyroidism (5). However, the study outcomes were assessed after a follow-up time of 12 months, which is relatively short for the event of clinical outcomes, and provided limited statistical power. Previously, a meta-analysis of observational studies showed that subclinical hypothyroidism was associated with an increased risk of coronary heart disease, particularly in individuals with a TSH concentration of 10 mIU/L or greater (4). Previous studies also concluded that the influence of subclinical hypothyroidism on lipids may depend on the degree of TSH elevation (33). Earlier, in a randomized, double-blind, placebo-controlled trial nested within the TRUST trial, including Swiss participants aged 65 years and over with mild subclinical hypothyroidism, systolic and diastolic heart function were found not to differ after treatment with levothyroxine compared with placebo (6), nor did carotid intima media thickness (CIMT) and maximum plaque thickness (36). In line with this, we observed clearer directionally consistent responses for the response of cardiometabolic biomarkers to levothyroxine treatment in the subgroup with TSH ≥10 mIU/L. Overall, levothyroxine treatment may not be beneficial for lipid metabolism, although certain subgroups, particularly those with TSH levels ≥10 mIU/L, may experience measurable improvements. One potential explanation is that individuals in the subgroup with baseline TSH ≥10 mIU/L are likely to have lower T4 levels and a more unfavorable lipid profile at baseline. More research is required to evaluate the clinical relevance of the potential improvements in lipid profile in those with TSH levels ≥10 mIU/L. Previous individual participant meta-analyses on the effect of cholesterol lowering on cardiovascular endpoints observed that for each 1.0 mmol/L reduction in LDL cholesterol, the annual rate of major cardiovascular events was reduced by approximately 20%, also in older people (37, 38). When extrapolating these results to those of our exploratory analyses, the observed potential levothyroxine-induced reduction of LDL cholesterol by 0.3 mmol/L in those with TSH levels ≥10 mIU/L would be associated with a reduced cardiovascular disease risk of about 6–7%. However, given the small sample size of the subgroup where a trend of possible benefit was observed (TSH ≥10 mU/L), future well-designed and sufficiently powered clinical trials are required to further corroborate these exploratory results.

Our study provided evidence supporting a null effect of levothyroxine on lipid metabolism in individuals with TSH <10 mIU/L, who represent the majority of those with subclinical hypothyroidism. These findings underscore the need for caution in overtreating individuals with levothyroxine with the goal of cardiovascular prevention within these groups. Our findings are supported by current guidelines, which suggest a more selective approach to levothyroxine initiation based on the magnitude of TSH elevations and symptomatology (27). However, the clinical relevance of the trends in potential lipid improvements observed in our study warrants further investigation. While the trends toward reductions in atherogenic lipids or ApoB-containing lipoprotein are promising, the extent to which these potentially favorable changes translate into long-term cardiovascular risk reduction remains uncertain.

One major strength of our study is that it is the first comprehensive post-hoc analysis of RCTs to investigate the effects of levothyroxine on detailed lipid profiles and metabolomic measures using standardized platform, with both approaches providing mutually supportive evidence. However, some limitations of the present study should also be acknowledged. First, the small sample size may have limited statistical power to detect significant effects, particularly after multiple-testing correction for the number of independent tests. Larger studies are needed to confirm these findings from our study. In addition, although we considered baseline statin use, changes in lipid-lowering therapy during follow-up were not considered, which may have influenced lipid levels at the study endpoint. Future studies should incorporate these factors to provide a more comprehensive understanding of levothyroxine's impact on lipid metabolism.

In conclusion, post-hoc analysis of previously performed RCTs indicates that levothyroxine treatment showed no clear cardiometabolic benefits in the general older population, although potentially favorable changes in decreased lipids and lipoproteins were observed for individuals with baseline TSH ≥10 mIU/L. Our findings support current guidelines that potential levothyroxine treatment in older adults with subclinical hypothyroidism should take into account baseline TSH concentrations, highlighting the importance of personalized treatment strategies and future well-designed clinical trials.

## Data Availability

Restrictions apply to the availability of some or all data generated or analyzed during this study to preserve patient confidentiality or because they were used under license. The corresponding author will on request detail the restrictions and any conditions under which access to some data may be provided.

## Clinical trial information

[ClinicalTrials.gov], identifier [NCT01660126] (TRUST); Netherlands Trial Register: NTR3851 (IEMO80+).
